# The Prognostic Role of Para-Aortic Lymph Node Metastasis in Patients with Resected Pancreatic Adenocarcinoma

**DOI:** 10.3390/cancers17213418

**Published:** 2025-10-24

**Authors:** Maximilian Brunner, Lena Kitzke, Anke Mittelstädt, Susanne Merkel, Georg F. Weber, Robert Grützmann, Christian Krautz

**Affiliations:** Department of General and Visceral Surgery, University Hospital Erlangen, Friedrich-Alexander-University (FAU), Krankenhausstraße 12, 91054 Erlangen, Germany; lena.kitzke@fau.de (L.K.); anke.mittelstaedt@uk-erlangen.de (A.M.); georg.weber@uk-erlangen.de (G.F.W.); robert.gruetzmann@uk-erlangen.de (R.G.); christian.krautz@uk-erlangen.de (C.K.)

**Keywords:** pancreatic ductal adenocarcinoma, paraaortal lymph node metastasis, survival

## Abstract

*Background:* This study aimed to evaluate the prognostic significance of para-aortic lymph node dissection (PALND) during pancreatic head resection and the impact of para-aortic lymph node metastasis (PALN+) on survival outcomes in patients with resected pancreatic ductal adenocarcinoma (PDAC). *Methods:* A retrospective analysis was conducted on 198 patients who underwent primary pancreatic head resection for PDAC at the University Hospital Erlangen between 2003 and 2022. Patients were stratified based on the presence or absence of PALND and PALN metastases, and their clinicopathological characteristics and survival outcomes were compared. *Results:* Of the 198 patients, 113 (57%) underwent additional PALND. PALND itself had no significant impact on overall survival (OS) or disease-free survival (DFS) compared to those without PALND. Among patients who underwent PALND, 17 (15%) had PALN metastases (PALN+). PALN+ patients exhibited significantly worse pathological features, including a higher rate of regional lymph node metastases (pN+), lymphovascular invasion (L1) and vascular invasion (V1). Survival analysis showed that PALN+ was associated with significantly poorer OS (8.7 vs. 29.3 months, *p* < 0.001) and DFS (3.8 vs. 17.0 months, *p* < 0.001). In multivariate analysis, PALN+ was confirmed as an independent prognostic factor for both OS (HR 1.9 [1.0–3.6], *p* = 0.035) and DFS (HR 2.2 [1.2–4.0], *p* = 0.006). *Conclusions:* While PALND does not impact survival outcomes in PDAC, it plays a crucial role in identifying PALN+ patients, who have significantly worse prognoses. PALN status should be integrated into clinical decision-making, particularly when considering intensified adjuvant therapy.

## Simple Summary

Pancreatic cancer remains one of the deadliest cancers, and even after surgery, many patients experience early recurrence and poor survival. During surgery for pancreatic cancer, removal and examination of para-aortic lymph nodes can help determine how far the cancer has spread. However, it is unclear whether removing these lymph nodes improves survival. This study examined patients who underwent pancreatic surgery with or without para-aortic lymph node dissection to assess its value. The results showed that removing these lymph nodes does not directly improve survival but helps identify patients whose cancer has spread further and who therefore have a worse prognosis. Recognizing these high-risk patients may help clinicians tailor follow-up treatments, such as intensified chemotherapy, and improve decision-making in patient care in the future.

## 1. Introduction

Pancreatic ductal adenocarcinoma of the head remains a highly aggressive malignancy with a poor prognosis. It is currently the fourth leading cause of cancer-related death in Europe and the third in the United States, with projections indicating it may become the second leading cause by 2030. Despite advancements in surgical techniques and systemic therapies, only 15–20% of patients present with resectable disease and the 5-year survival rate remains low, ranging from 5% to 30% [1,2,3]. While adjuvant chemotherapy has improved survival, long-term outcomes are still limited due to late-stage diagnosis and high recurrence rates [4,5,6,7].

Surgical resection, primarily pancreatoduodenectomy (PD), remains the only potentially curative treatment for PDAC of the head. The extent of lymphadenectomy in pancreatic cancer surgery has been standardized, with international consensus on which lymph node (LN) stations should be included. According to the International Study Group for Pancreatic Surgery (ISGPS), standard lymphadenectomy encompasses peripancreatic, hepatic, pyloric and mesenteric lymph nodes [8]. However, the role of para-aortic lymph node (PALN, station 16, lymph nodes from the interaortocaval region, located between the inferior vena cava and the aorta, extending from the right crus of the diaphragm as the cranial border to the level of the inferior mesenteric artery as the caudal border) dissection remains controversial [9,10,11,12,13,14,15]. PALN metastases are classified as distant disease (pM1) by the Union for International Cancer Control (UICC) and the Japanese Pancreas Society, raising concerns about their impact on prognosis and surgical decision-making [16,17].

Reported rates of PALN involvement in resectable PDAC of the head range from 12% to 21% [9,10,11,12,13,14,15]. Some studies associate PALN metastases with reduced overall survival, leading some surgeons to consider them a contraindication for resection [15]. However, other reports suggest that PALN status is not an independent prognostic factor and should not preclude surgical intervention [9,10,11]. Consequently, different surgical approaches exist: (1) omitting PALN dissection, (2) performing PALN sampling with intraoperative frozen section analysis and aborting resection if positive or (3) routinely including PALN dissection in standard pancreatic surgery, regardless of preoperative findings.

CT remains the gold standard for preoperative staging of periampullary tumors, but its sensitivity in detecting PALN metastases remains debated [10,18]. As many patients with resectable PDAC of the head already present with locally advanced disease, using PALN status for treatment stratification may lead to unnecessary treatment restrictions.

This study aims to investigate the prognostic relevance of para-aortic lymph node dissection (PALND) during pancreatic head resection and the impact of para-aortic lymph node metastasis (PALN+) on survival outcomes in a consecutively treated cohort of patients with resected pancreatic head ductal adenocarcinoma (PDAC).

## 2. Material and Methods

For this retrospective analysis, prospectively collected data from the Erlangen Cancer Registry of the Department of Surgery were utilized. We included all adult patients diagnosed with pancreatic ductal adenocarcinoma (PDAC) who underwent pancreatic head resection between 1 January 2003 and 31 December 2022 at the Surgical Department of the University Hospital Erlangen. Only patients with primarily resectable PDAC of the pancreatic head were considered. Exclusion criteria included neoadjuvant therapy, ampullary carcinoma, pT4 category, postoperative R2 (macroscopically incomplete resection) resection status and the presence of distant metastases other than para-aortic lymph node (PALN) metastases.

Clinical and pathological data were retrieved from the hospital’s clinical information system, while survival data were obtained from the Erlangen Cancer Registry. Histopathological classification was based on the TNM classification of malignant tumors by the Union for International Cancer Control (UICC), according to the 8th edition (2017) [16]. Postoperative morbidity was assessed using the Clavien–Dindo classification, with major morbidity defined as Clavien–Dindo ≥ 3 [19].

This retrospective study was approved by the local ethics committee (22-165-Br).

### 2.1. Surgical and Postoperative Procedures

All pancreatic head resections were performed by highly experienced visceral surgeons specializing in pancreatic surgery. A standardized surgical approach with oncologically appropriate lymphadenectomy was applied in all cases of PDAC [8,20]. Extended resection procedures, including vascular or multivisceral resections, were performed if necessary to achieve an R0 (microscopically margin-negative resection) status. Arterial resections were only considered in exceptional cases. If intraoperative findings confirmed unresectable disease, such as liver metastases or peritoneal carcinomatosis, the procedure was aborted.

Para-aortic lymph node dissection (PALND) was performed at the surgeon’s discretion, generally when intraoperative findings suggested possible nodal involvement or when preoperative imaging was inconclusive. The procedure involved en bloc removal of lymph nodes from the interaortocaval region, specifically station 16, located between the inferior vena cava and the aorta, extending from the right crus of the diaphragm as the cranial border to the level of the inferior mesenteric artery as the caudal border. PALND aimed to enhance staging accuracy and assess the prognostic significance of para-aortic lymph node metastases (PALN+). Dissected lymph nodes were sent for histopathological analysis, but intraoperative frozen section analysis was not routinely performed. It was selectively used in cases where PALN status might influence the decision to proceed with or abandon resection. However, the final determination of resectability was based on a combination of preoperative imaging, intraoperative findings and the absence of distant metastases.

Postoperatively, all cases were discussed in a multidisciplinary tumor board, taking into account the full histopathological assessment. Adjuvant chemotherapy was recommended for all eligible patients, provided their overall condition permitted systemic treatment. Chemotherapy regimens varied over time and included gemcitabine, gemcitabine plus nab-paclitaxel or FOLFIRINOX (5-fluorouracil, leucovorin, irinotecan, oxaliplatin). Routine follow-up care was advised for all patients, including regular imaging (CT scans) and monitoring of CA19-9 tumor markers.

### 2.2. Statistical Analysis

Data analysis was performed using SPSS^®^ Version 28 (IBM, Armonk, NY, USA). Results are presented as *n* (%) or median (interquartile range, IQR). Comparisons of continuous and ordinal variables were conducted using the Kruskal–Wallis test, while categorical data were analyzed with the Chi-square test. A *p*-value ≤ 0.05 was considered statistically significant.

Overall survival (OS) was defined as the time from surgery to death or last follow-up, while disease-free survival (DFS) was measured from surgery to death, recurrence, or last follow-up. Time intervals are presented in months. Survival curves were generated using the Kaplan–Meier method and compared using the log-rank test. Prognostic factors for survival were assessed through univariate and multivariate analyses. Variables with a *p* ≤ 0.05 in univariate analysis were included in multivariate analysis using the Cox regression model.

## 3. Results

### 3.1. Patient Characteristics

The study cohort comprised 198 patients with PDAC who underwent pancreatic head resection between 2003 and 2022. Among them, 113 (57%) underwent additional para-aortic lymph node dissection (PALND). Patient characteristics were comparable between groups (Table 1). Morbidity and major morbidity were not increased in the PALND group (42% vs. 36%, *p* = 0.553, and 22% vs. 19%, *p* = 0.592, respectively).

Histological analysis revealed para-aortic lymph node metastases (PALN+) in 17 patients (15%). PALN+ patients had a significantly higher incidence of regional lymph node metastases (pN+) (100% vs. 59%, *p* = 0.002), lymphangiosis involvement (L1) (76% vs. 28%, *p* < 0.001) and vascular invasion (V1) (29% vs. 8%, *p* = 0.026) compared to PALN− patients. Other patient and histopathological characteristics showed no significant differences between the groups (Table 1).

### 3.2. Outcome Parameters

Recurrence occurred in 57% of the cohort, predominantly as metastatic disease. The recurrence rate and pattern showed no significant differences between patients with and without PALND, as well as between PALN+ and PALN− groups (Table 2).

**Figure 1:**
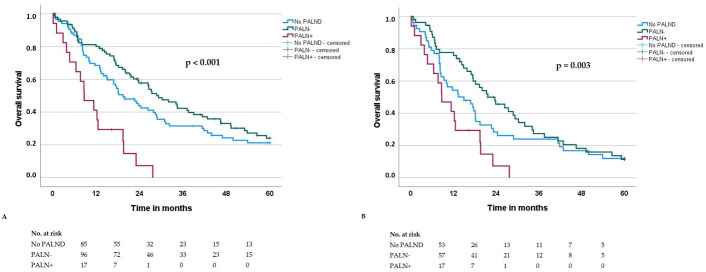
Overall survival of patients with resected pancreatic head ductal adenocarcinoma, stratified by those without para-aortic lymph node dissection (No PALND), those with PALND and without para-aortic lymph node metastasis (PALN−) and those with PALND and para-aortic lymph node metastasis (PALN+). (**A**): All patients (*n* = 199); (**B**): Patients with locoregional lymph node metastasis (pN+) (*n* = 127).

**Figure 2:**
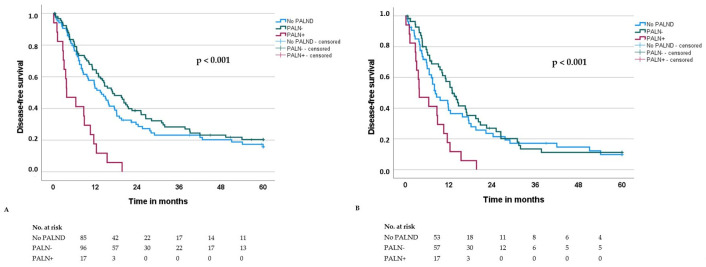
Disease-free survival of patients with resected pancreatic head ductal adenocarcinoma, stratified by those without para-aortic lymph node dissection (No PALND), those with PALND and without para-aortic lymph node metastasis (PALN−) and those with PALND and para-aortic lymph node metastasis (PALN+). (**A**): All patients (*n* = 199); (**B**): Patients with locoregional lymph node metastasis (pN+) (*n* = 127).

The median overall survival (OS) and disease-free survival (DFS) for the entire cohort were 23.1 and 14.1 months, respectively. OS and DFS did not significantly differ between patients with and without PALND (OS: 26.4 vs. 19.5 months, *p* = 0.696; DFS: 14.4 vs. 13.8 months, *p* = 0.883). However, both OS and DFS were significantly shorter in PALN+ patients compared to PALN− patients (OS: 8.7 vs. 29.3 months, *p* < 0.001; DFS: 3.8 vs. 17.0 months, *p* < 0.001) (Table 2; Figure 1A and Figure 2A).

### 3.3. Prognostic Factors for Overall Survival After Surgery (OS) and Disease-Free Survival (DFS) in the Whole Patient Cohort (n = 199)

Potential prognostic factors for overall survival (OS) and disease-free survival (DFS) after PDAC resection are presented in Table 3 and Table 4, respectively.

In univariate analysis, twelve factors were significantly associated with worse OS: age >70 years, ASA III status, locally advanced tumors (pT3), lymph node metastasis (pN+), R1 status, poor differentiation (G3), perineural invasion (Pn1), lymphangiosis involvement (L1), vascular invasion (V1), PALN+, major morbidity and absence of adjuvant chemotherapy. Multivariate analysis identified the following as independent predictors of worse OS: age > 70 years (HR 1.9 [1.3–2.8], *p* = 0.002), lymph node metastasis (HR 2.6 [1.7–4.2], *p* < 0.001), R1 status (HR 2.8 [1.4–5.5], *p* = 0.003), G3 differentiation (HR 1.7 [1.0–2.7], *p* = 0.035), vascular invasion (HR 2.6 [1.5–4.4], *p* < 0.001), PALN+ (HR 1.9 [1.0–3.6], *p* = 0.035) and lack of adjuvant chemotherapy (HR 2.5 [1.6–3.8], *p* < 0.001) (Table 3).

For DFS, univariate analysis identified ten factors significantly associated with worse prognosis: age > 70 years, locally advanced tumors (pT3), lymph node metastasis (pN+), poor differentiation (G3), perineural invasion (Pn1), lymphangiosis involvement (L1), vascular invasion (V1), PALN+, major morbidity and absence of adjuvant chemotherapy. Multivariate analysis confirmed age >70 years (HR 1.6 [1.1–2.2], *p* = 0.018), lymph node metastasis (HR 1.9 [1.3–3.0], *p* = 0.002), G3 differentiation (HR 1.6 [1.0–2.4], *p* = 0.034), vascular invasion (HR 1.8 [1.1–3.0], *p* = 0.020), PALN+ (HR 2.2 [1.2–4.0], *p* = 0.006) and lack of adjuvant chemotherapy (HR 2.1 [1.4–3.1], *p* < 0.001) as independent predictors of worse DFS (Table 4).

## 4. Discussion

The present study analyzed the prognostic significance of para-aortic lymph node dissection (PALND) and para-aortic lymph node metastases (PALN+) in patients undergoing pancreatic head resection for PDAC. Our findings indicate that while PALND itself does not impact overall survival (OS) or disease-free survival (DFS), the presence of PALN metastases (PALN+) is a strong independent predictor of worse outcomes.

In our cohort, para-aortic lymph node metastasis was present in 15% of the patients who underwent PALND. This is in line with previously reported prevalences of para-aortic lymph node metastasis, which range from 12% to 21% [9,10,11,12,13,14,15].

A key observation was that PALN+ patients had significantly more frequent regional lymph node metastases (pN+), lymphangiosis involvement (L1), and vascular invasion (V1), suggesting that PALN metastases are associated with a more advanced tumor stage and/or aggressive tumor biology. This is consistent with previous studies that have reported an increased lymph node ratio (LNR) and a correlation between PALN+ and systemic disease progression [9].

Despite the lack of survival benefits from PALND itself, our findings highlight the prognostic value of PALN status. Patients with PALN+ had significantly lower OS (8.7 vs. 29.3 months) and DFS (3.8 vs. 17.0 months) compared to PALN− patients. Simultaneously, morbidity was not affected by PALND suggesting it to be safe. This underscores the importance of PALN assessment for risk stratification. Patients undergoing PALND and those who did not both comprise individuals with PALN+ and PALN– status, although the precise distribution of PALN involvement in the non-PALND group remains unknown. This likely explains the lack of significant differences in outcomes between these groups. In contrast, stratification by PALN status (PALN– vs. PALN+) reflects a biologically meaningful parameter of tumor dissemination. PALN+ indicates systemic spread beyond the regional lymphatic basin, which may account for the observed significant differences in recurrence, overall survival (OS), and disease-free survival (DFS).

These results are consistent to a large meta-analysis from 2016 including 13 studies and 2141 patients showing a significantly worse survival outcome in patients with involved PALN. Moreover, similar to our results, this meta-analysis confirmed that the impact of PALN+ on survival is still present considering only pN+-patients (Figure 1B and Figure 2B) [14].

Multivariate analysis confirmed PALN+ as an independent prognostic factor for both OS and DFS, alongside established parameters such as age >70 years, pN+, poor differentiation (G3), vascular invasion (V1), R1 resection and absence of adjuvant chemotherapy. These results underscore the value of PALN as a risk stratification marker and reaffirm the importance of adjuvant therapy and achieving R0 resections for improved survival.

A subset of patients in our cohort experienced recurrence within six months despite macroscopically complete resection, consistent with the concept of “biological R2” disease [21]. This phenomenon reflects aggressive tumor biology rather than technical factors and may explain why PALN+ patients—serving as an indicator of systemic disease spread and a higher likelihood of biological R2—exhibit significantly worse outcomes.

Given the significant prognostic role of PALN involvement, some authors have proposed that PALN+ may reflect systemic disease and thus represent a contraindication for surgical resection [15]. However, most suggest that surgical treatment remains justified in selected patients, particularly when embedded in a multimodal treatment strategy [9,10,11]. Several studies have indicated that even in the presence of PALN metastases, patients may derive a survival benefit from pancreatic resection compared to exploration or palliative double bypass surgery [11,22]. Notably, Kim et al. demonstrated in their analysis of PDAC patients undergoing pancreatic head resection that the administration of adjuvant chemotherapy may mitigate the negative prognostic impact of PALN involvement, resulting in survival outcomes comparable to those of patients with regional lymph node metastases [23].

In contrast, some studies have reported no or only minor survival differences between PALN+ and PALN− patients, both in unselected PDAC cohorts and in pN+ subgroups [9,10,24]. Possible explanations for these discrepancies include differences in patient selection, tumor biology, surgical technique, and institutional treatment protocols. In some cohorts, PALN involvement may reflect isolated nodal spread, whereas in others it may indicate advanced systemic disease. Variations in the extent of lymphadenectomy, use of adjuvant therapies, evolving treatment standards, as well as limited sample sizes and statistical power, may further contribute to heterogeneity across studies.

The high recurrence rate in our cohort (57%), predominantly distant metastases, further reflects the aggressive nature of PDAC. Importantly, recurrence patterns were not influenced by PALND, as rates of local recurrence, including PALN recurrences, were comparable between groups.

Our findings support routine intraoperative sampling of para-aortic lymph nodes for improved prognostic stratification. As PALND is technically simple and not associated with increased morbidity, it may identify PALN+ patients who could benefit from more aggressive systemic treatment strategies. Even though existing data on morbidity show no difference, information on postoperative recovery and quality of life after PALND is limited. Only Safi et al. reported on postoperative hospital stay, finding no significant difference between patients with and without PALND (23 vs. 20.5 days) [10]. Data specifically addressing quality of life after PALND are not available.

Future research should clarify whether neoadjuvant approaches can improve outcomes in PALN+ patients, as suggested by emerging treatment algorithms [25]. However, more reliable preoperative diagnostic tools will be necessary to identify PALN involvement prior to surgery [10,18].

This study is limited by its retrospective design, its limited patient number—especially regarding the long observation period of 20 years—and potential selection bias, as PALND was not performed in all patients. Additionally, variations in adjuvant treatment regimens over the study period could have influenced survival outcomes. Prospective trials are needed to clarify the optimal treatment approach for PALN+ patients and to determine whether extended lymphadenectomy provides any therapeutic advantage beyond prognostic stratification.

## 5. Conclusions

In summary, PALND does not influence survival outcomes in PDAC but serves as an important diagnostic tool for identifying PALN+ patients, who have significantly worse OS and DFS. PALN status should be considered in clinical decision-making, particularly regarding the use of intensified adjuvant therapy. Future research should focus on refining treatment strategies for PALN+ patients to improve their currently poor prognosis.

## Figures and Tables

**Table 1:** Clinicopathological characteristics of patients undergoing pancreatic head resection for pancreatic ductal adenocarcinoma, stratified by those with and without para-aortic lymph node dissection (No PALND vs. PALND) and those with and without para-aortic lymph node metastasis (PALN− vs. PALN+).

	Pancreatic Head Resection Without PALND	Pancreatic Head Resection with PALND	*p*-Value (No PALND vs. PALND)	Pancreatic Head Resection with PALND and PALN−	Pancreatic Head Resection with PALND and PALN+	*p*-Value (PALN− vs. PALN+)
**Number**	85	113	-	96	17	-
**Age (years), median (IQR)**	71 (12)	72 (15)	0.620	70 (16)	75 (9)	0.178
**Sex,** ***n*** **(%)** **Female** **Male**	44 (52) 41 (48)	45 (40) 68 (60)	0.113	40 (42) 56 (58)	5 (29) 12 (71)	0.426
**ASA,** ***n*** **(%)** **I** **II** **III** **Unknown**	4 (5) 40 (47) 38 (45) 3 (3)	6 (5) 60 (53) 45 (40) 2 (2)	0.786	6 (6) 50 (52) 38 (40) 2 (2)	0 (0) 10 (59) 7 (41) 0 (0)	0.817
**pT category,** ***n*** **(%)** **pT1** **pT2** **pT3**	13 (15) 45 (53) 27 (32)	16 (14) 71 (63) 26 (23)	0.336	16 (17) 58 (60) 22 (23)	0 (0) 13 (77) 4 (24)	0.203
**pN category,** ***n*** **(%)** **pN0** **pN+**	32 (38) 53 (62)	39 (35) 74 (65)	0.657	39 (41) 57 (59)	0 (0) 17 (100)	**0.002**
**R classification,** ***n*** **(%)** **R0** **R1**	76 (89) 9 (11)	109 (96) 4 (4)	0.079	93 (97) 3 (3)	16 (94) 1 (6)	1.000
**Grading,** ***n*** **(%)** **G1/2** **G3**	25 (29) 60 (71)	35 (31) 78 (69)	0.876	33 (34) 63 (66)	2 (12) 15 (88)	0.087
**Perineural invasion (Pn),** ***n*** **(%)** **0** **1**	22 (26) 63 (74)	27 (24) 86 (76)	0.868	25 (26) 71 (74)	2 (12) 15 (88)	0.239
**Lymphangiosis (L),** ***n*** **(%)** **0** **1**	60 (71) 25 (29)	73 (65) 40 (35)	0.445	69 (72) 27 (28)	4 (24) 13 (76)	**<0.001**
**Vascular invasion (V), *n* (%)** **0** **1**	72 (84) 14 (16)	100 (88) 13 (12)	0.403	88 (92) 8 (8)	12 (71) 5 (29)	**0.026**
**Major morbidity,** ***n*** **(%)**	19 (22)	21 (19)	0.592	18 (19)	3 (18)	1.000
**Adjuvant chemotherapy,** ***n*** **(%)**	50 (58)	70 (62)	0.663	59 (62)	11 (65)	1.000

Data are presented as number (%) or as median (interquartile range, IQR). PALND = Peri-Aortal Lymph Node Dissection, PALN = Peri-Aortal Lymph Nodes; ASA = American Society of Anesthesiologists classification; BMI = Body Mass Index. Bold = significant.

**Table 2:** Outcome parameter of patients undergoing pancreatic head resection for pancreatic ductal adenocarcinoma, stratified by those with and without para-aortic lymph node dissection (No PALND vs. PALND) and those with and without para-aortic lymph node metastasis (PALN− vs. PALN+).

	Pancreatic Head Resection Without PALND (*n* = 85)	Pancreatic Head Resection with PALND (*n* = 113)	*p*-Value (No PALND vs. PALND)	Pancreatic Head Resection with PALND and PALN− (*n* = 96)	Pancreatic Head Resection with PALND and PALN+ (*n* = 17)	*p*-Value (PALN− vs. PALN+)
**Overall survival (months), median (SD)**	19.5 (3.2)	26.4 (2.9)	0.696	29.3 (4.0)	8.7 (2.5)	**<0.001**
**Recurrence, *n* (%)**	48 (57)	64 (57)	1.000	53 (55)	11 (65)	0.598
**Recurrence pattern,** ***n*** **(%)** **Metastatic** **Locoregional** **Both**	26 (54) 7 (15) 15 (31)	42 (66) 5 (8) 17 (27)	0.400	34 (64) 5 (9) 14 (26)	8 (73) 0 (0) 3 (27)	0.685
**Disease-free survival (months), median (SD)**	13.8 (1.9)	14.4 (1.8)	0.883	17.0 (2.5)	3.8 (2.0)	**<0.001**

Data are presented as number (%) or as median (Standard Deviation, SD). Bold = significant.

**Table 3:** Prognostic factors for overall survival (OS) in patients with resected pancreatic head ductal adenocarcinoma.

		Univariate		Multivariate		
	*n*	Median OS	*p*	HR	95% CI	*p*-Value
**Age** **≤70 years** **>70 years**	94 104	32.1 17.4	**<0.001**	**1.9**	**1.3–2.8**	**0.002**
**Sex** **Female** **Male**	89 109	23.8 23.1	0.946	-	-	-
**ASA (*****n*** = 194) * **I/II** **III**	111 83	26.6 17.0	**0.014**	1.3	0.9–1.8	0.232
**pT category** **pT1/pT2** **pT3**	145 53	26.6 15.1	**0.016**	1.3	0.8–2.0	0.260
**pN category** **pN0** **pN+**	71 127	44.1 17.5	**<0.001**	**2.6**	**1.7–4.2**	**<0.001**
**R classification** **R0** **R1**	185 13	23.7 10.3	**0.031**	**2.8**	**1.4–5.5**	**0.003**
**Grading** **G1/G2** **G3**	60 138	37.7 18.3	**<0.001**	**1.7**	**1.0–2.7**	**0.035**
**Perineural invasion** **Pn0** **Pn1**	49 149	41.4 19.7	**0.006**	1.0	0.6–1.6	0.979
**Lymphangiosis** **L0** **L1**	133 65	27.4 17.0	**<0.001**	1.1	0.7–1.7	0.751
**Vascular invasion** **V0** **V1**	172 26	24.4 12.3	**0.007**	**2.6**	**1.5–4.4**	**<0.001**
**PALN(D)** **No PALND** **PALN−** **PALN+**	85 96 17	19.5 29.4 8.7	**<0.001**	**1.0** **0.8** **1.9**	**0.6–1.2** **1.0–3.6**	**0.035**
**Major morbidity** **Yes** **No**	40 158	15.1 26.6	**<0.001**	1.0	0.6–1.6	0.979
**Adjuvant chemotherapy** **Yes** **No**	120 78	26.6 17.0	**0.012**	**2.5**	**1.6–3.8**	**<0.001**

ASA = American Society of Anesthesiologists classification. * missing data. Bold = significant.

**Table 4:** Prognostic factors for disease-free survival (DFS) in patients with resected pancreatic head ductal adenocarcinoma.

		Univariate		Multivariate		
	*n*	Median OS	*p*	HR	95% CI	*p*-Value
**Age** **≤70 years** **>70 years**	94 104	18.2 10.1	**0.001**	**1.6**	**1.1–2.2**	**0.018**
**Sex** **Female** **Male**	89 109	13.8 14.4	0.759	-	-	-
**ASA (*****n*** = 194) * **I/II** **III**	111 83	14.9 13.0	0.063	-	-	-
**pT category** **pT1/pT2** **pT3**	145 53	16.0 9.0	**0.023**	1.3	0.9–2.0	0.130
**pN category** **pN0** **pN+**	71 127	21.5 10.6	**<0.001**	**1.9**	**1.3–3.0**	**0.002**
**R classification** **R0** **R1**	185 13	14.6 10.1	0.120	-	-	-
**Grading** **G1/G2** **G3**	60 138	21.2 11.7	**<0.001**	**1.6**	**1.0–2.4**	**0.034**
**Perineural invasion** **Pn0** **Pn1**	49 149	23.7 12.3	**0.003**	1.2	0.8–1.9	0.434
**Lymphangiosis** **L0** **L1**	133 65	16.6 9.1	**<0.001**	1.3	0.8–1.9	0.252
**Vascular invasion** **V0** **V1**	172 26	14.7 8.7	**0.038**	**1.8**	**1.1–3.0**	**0.020**
**PALN** **No PALND** **PALN−** **PALN+**	85 96 17	13.8 17.0 3.8	**<0.001**	**1.0** **0.8** **2.2**	**0.6–1.2** **1.2–4.0**	**0.006**
**Major morbidity** **Yes** **No**	40 158	7.0 15.5	**0.003**	1.1	0.7–1.8	0.600
**Adjuvant chemotherapy** **Yes** **No**	120 78	15.5 8.0	**0.015**	**2.1**	**1.4–3.1**	**<0.001**

ASA = American Society of Anesthesiologists classification. * missing data. Bold = significant.

## Data Availability

All data generated or analyzed during this study are included in this published article.
